# The Impact of Employees’ Psychological Capital on Innovative Work Behavior: The Chain Mediating Effect of Knowledge Donating and Knowledge Collecting

**DOI:** 10.3389/fpsyg.2021.761399

**Published:** 2021-12-14

**Authors:** Weijing Chen, Xiaoyun Zhu, Shan Sun, Shudi Liao, Zhiwen Guo

**Affiliations:** ^1^Department of Human Resource Management, Business School, Hubei University, Wuhan, China; ^2^Hubei Center for Talent Development Strategy and Policy Studies, Wuhan, China; ^3^Department of Psychology, School of Education, Hubei University, Wuhan, China

**Keywords:** innovative work behavior, knowledge sharing, knowledge donating, knowledge collecting, psychological capital

## Abstract

This study aimed to test the mediating role of knowledge sharing, which includes two central processes of knowledge collecting and knowledge donating, in the relationship of psychological capital and innovative work behavior (IWB). The proposed theoretical framework was based on the theory of reasoned action and social exchange theory. In a field study, using a research sample of 345 valid leader-subordinate matching data, we tested three competitive models to explore the different mediating effects of knowledge collecting and donating. Results indicated that knowledge donating and knowledge collecting played a chain mediating role between psychological capital and IWB, and the independent mediating effect of knowledge collecting was also significant. From the perspective of knowledge sharing, the present study deeply analyzes the psychological processing mechanism of psychological capital on IWB, confirms the positive significance of knowledge donating at the individual level, and provides a new perspective for organizations to promote employees’ knowledge sharing and stimulate their IWB.

## Introduction

Innovation is the inevitable choice for the survival and development of organizations. As the main body of organizational innovation, employees’ innovation is closely related to organizational innovation. Innovative work behavior (IWB) is a comprehensive index to measure employees’ innovation (Scott and Bruce, 1994; Janssen, 2000; Yuan and Woodman, 2010; Anderson et al., 2014), which focuses on the whole innovation process and represents the continuous, dedicated, and sincere efforts of employees (Agarwal, 2014; Akram et al., 2020). However, in reality, not all employees are willing to engage in innovation activities unconditionally. Innovation activities are usually accompanied by high risk and complexity (Carmeli and Schaubroeck, 2007). The implementation of new ideas may face failure or may be rejected and criticized by other organization members. Only a strong internal drive can help individuals stick to innovation activities to the end. Therefore, exploring the internal driving force of employees’ IWB and opening the “black box” of its mechanism has become an important research topic in the field of organizational psychology.

Psychological capital is a positive psychological state and mental energy built during the process of individual growth and development (Baron et al., 2016), as while as an important psychological resource that promotes individual growth and performance (Newman et al., 2014; Philipp et al., 2019). Research suggests that psychological capital helps to stimulate employees’ innovative vitality (Qiu et al., 2015; Guo et al., 2019; Gao et al., 2020), which not only has a direct impact on innovation, but also plays an indirect role through a certain intermediary mechanism. Focusing on the internal mechanism of how psychological capital affects employees’ IWB, scholars have conducted multi-angle discussions from the individual perspectives of mastery-oriented mindset (Luthans et al., 2011), job control (Yan et al., 2020), job satisfaction and organizational commitment (Tang et al., 2019), the organizational context perspectives of entrepreneurial leadership (Newman et al., 2018), and organizational innovation climate (Hsu and Chen, 2017). Innovation is a process in which individuals realize the spillover effect and value-added of knowledge by using and sharing knowledge. This is a mutually beneficial process of productive interaction between individuals and organizations. If we pay attention to the internal impact of psychological capital on IWB from the perspective of knowledge aggregation and two-way flow, it may provide a new perspective for us to understand the relationship between them.

As an important way to promote knowledge accumulation and flow, knowledge sharing has a positive impact on employees’ innovation ability and innovation behavior (Kim and Lee, 2013; Akhavan and Hosseini, 2016; Pian et al., 2019; Zhu et al., 2019; Akram et al., 2020; Vandavasi et al., 2020; Asurakkody and Kim, 2020; Yasir et al., 2021). Especially in the Information Age, social networking sites use digital and new media technology to provide a more convenient and highly interactive platform for individuals and groups, so as to play the role of knowledge to a greater extent (Kim et al., 2015). At the same time, we need to pay attention to the factors affecting employees’ willingness to participate in knowledge sharing, that is, the driving force of their participation in knowledge sharing, in which psychological capital is an important factor (Qiu et al., 2015; Wu and Lee, 2017; Zhang et al., 2017). The process of knowledge sharing is generally considered to comprise two aspects: contributing and obtaining knowledge (Weggeman, 2000; Oldenkamp, 2001; Ardichvili et al., 2003). Hooff and Ridder (2004), developers of the widely used knowledge sharing scale, pointed out that knowledge sharing is a process in which individuals mutually exchange knowledge and jointly create new knowledge, including two central processes: knowledge collecting and knowledge donating. Knowledge collecting is consulting colleagues in order to get them to share their intellectual capital; and knowledge donating is communicating to others what one’s personal intellectual capital is (Hooff and Ridder, 2004). For individuals, knowledge donating is the output of knowledge, and knowledge collecting is the acquisition of knowledge. Organizations usually encourage employees to actively participate in knowledge donating, because it is conducive to the integration and utilization of knowledge resources, the optimization and improvement of knowledge reserves, and then conducive to the improvement of organizational innovation ability (Spencer, 2003; Phene et al., 2006; Tassabehji et al., 2019). However, from the perspective of employees, they commonly experience thoughts of retaining knowledge to protect their own advantages and status. Bock et al. (2005) have pointed out that when an employee believes that choosing to share knowledge will cause him/her to lose his/her unique value within the organization, he/she will not readily share his/her knowledge with others. Therefore, what we often see in the organization is that employees are not keen on knowledge donating compared with knowledge collecting. What could I benefit from knowledge donating, this is the major concern of employees.

How to effectively promote knowledge sharing has always been one of the most important issues in the field of knowledge management (Zhang and Jiang, 2015; Obrenovic et al., 2020), among which how to promote knowledge donating is a more challenging issue. A few existing studies have paid attention to the independent role of knowledge collecting and knowledge donating, which are usually regarded them as two parallel variables (Kim and Lee, 2013; Akhavan and Hosseini, 2016; Kmieciak, 2020). In the process of knowledge exchange, collecting or donating knowledge will make individuals obtain different feelings and feedback, which will affect the subsequent knowledge donating or collecting behavior. According to the theory of reasoned action (TRA), an individual’s behavior is the final choice made through rational thinking after integrating his or her own value judgment, social norms, and expectations of others (Fishbein and Ajzen, 1975). And based on the social exchange theory (SET; Homans, 1958), especially the reciprocity norm (Gouldner, 1960), both sides of the exchange tend to reward each other with similar behavior, so there may be a correlation between knowledge collecting and donating, which has been confirmed by some studies (Hooff and Ridder, 2004; Hussein et al., 2016). However, their interaction and its impact on IWB have not been involved.

The present study draws from TRA and SET and analyzes the internal mechanism of the impact of psychological capital on IWB through the two central processes of knowledge sharing (knowledge collecting and knowledge donating) from an interacting perspective. We construct three competitive models: psychological capital creates conditions for knowledge collecting by promoting individual knowledge donating and then affects IWB; psychological capital stimulates individual knowledge collecting, makes them give back through knowledge donating, and then affects IWB; and psychological capital affects IWB through parallel knowledge collecting and knowledge donating. Through the analysis and verification of these hypotheses, we reveal the impact mechanism of individual knowledge inflow and outflow on employees’ IWB under the influence of psychological capital and explore the effective path to stimulate employees’ IWB.

## Literature Review and Hypotheses

### Psychological Capital and IWB

Innovation activities have high risks (Carmeli and Schaubroeck, 2007), which means that employees need strong internal support to participate in innovation, that is, the belief that they have the ability to produce creative results. Psychological capital is an individual’s positive internal trait and psychological state. The higher the psychological capital, the stronger the individual’s belief in his or her own creativity. Previous studies have proved that employees with high psychological capital usually perform well in terms of organizational capability, OCB, performance, and innovation (Luthans et al., 2007; Avey et al., 2011; Qiu et al., 2015; Slåtten et al., 2019). Luthans et al. (2003) divided psychological capital into four dimensions: self-efficacy, hope, optimism, and resilience. Employees with high psychological capital are more enthusiastic and energetic, full of curiosity and exploration, more willing to think and accept new ideas, which are important conditions to promote higher willingness and overall ability to innovate (Luthans et al., 2011; Tsegaye et al., 2020). Moreover, because of the uncertainty of external environments and its own breakthrough characteristics, innovation is often accompanied by high failure risk. High psychological capital means high hope and self-efficacy (Luthans et al., 2007), so that employees will pay more attention to the positive aspects of innovation and are more easily to recover confidence when they encounter risks or failures and then actively seek breakthroughs and improvements (Luthans et al., 2003; Carmeli, 2006; Cao, 2015; Andersson et al., 2020). Therefore, psychological capital is an important source and driving force of IWB (Guo et al., 2019; Tang et al., 2019; Gao et al., 2020; Yan et al., 2020). Following from this, our first hypothesis is as follows:

*Hypothesis 1:* Psychological capital is positively related to IWB.

### Psychological Capital and Knowledge Sharing

Individual’s different cognition of self will also affect their internal motivation and willingness to share knowledge to a great extent (McAllister, 1995). Previous studies have found that psychological capital is an important factor to promote employees’ knowledge sharing (Qiu et al., 2015; Wu and Lee, 2017; Zhang et al., 2017), which has a prominent impact on employees’ knowledge collecting and knowledge donating. Based on TRA, attitude and perception of the outside world will affect individual behavior. Therefore, in the face of organizational encouragement and their own demand for sharing knowledge, individual psychological capital will play a better driving role in knowledge collecting and donating behavior. In terms of knowledge collecting, employees with high psychological capital have positive psychological characteristics and external behaviors, which are more willing to communicate and integrate with the group. Communication is the basis and premise of all sharing (Bogler and Somech, 2019; He et al., 2019) and can provide channels and conditions for employees to collect knowledge. In terms of knowledge donating, knowledge donating is accompanied by potential risks: as the knowledge reserves of employees are limited, the process of donating knowledge will remove their exclusive rights to knowledge, diluting the unique skills and competitive advantages that employees possess. However, psychological capital is closely related to psychological security (Darvishmotevali and Ali, 2020). Employees with high psychological capital have a stronger belief in the roles of personal ability and organizational support and have a lower perception of the threat posed by knowledge sharing to individuals. They are good at objective and flexible positive attribution and are easy to perceive the positive aspects of interpersonal trust within the organization, which makes them more willing to share their skills and experience with other colleagues (Luthans and Youssef, 2004). In addition, a higher sense of self-efficacy and hope will also drive individuals to have a higher sense of self-belonging and self-realization and make individuals realize that sharing knowledge and experience with others is an affirmation and promotion of themselves, so as to strengthen the internal motivation and willingness of individuals to donate knowledge. Finally, the realization of knowledge collecting and donating will always encounter certain obstacles, such as communication effectiveness obstacles (influence of communication technology and tools) and interpersonal trust. Employees with high psychological capital have a stronger sense of self-efficacy, can better perceive the trust and recognition they have obtained, help to break the communication barriers and participate in sharing more efficiently (Bogler and Somech, 2019).

Although as active processes of knowledge sharing (Hooff and Ridder, 2004), both knowledge collecting and knowledge donating may be affected by psychological capital, the degree of impact may be different due to their nature. Knowledge collecting constitutes enabling oneself to profit from others’ intellectual capital, which, in economically rational terms, means that the benefits outweigh the costs (Hooff and Weenen, 2004); therefore, employees are more likely to accept and actively participate in this process. Donating knowledge constitutes sharing one’s intellectual capital with others, in which the costs may significantly outweigh the benefits (Hooff and Weenen, 2004), which needs to overcome the sense of threat caused by the loss of exclusive rights to knowledge; it therefore needs stronger driving forces, such as the support of psychological capital. Hence, we believe that psychological capital will have a stronger impact on knowledge donating than on knowledge collecting, and we propose the following hypothesis:

*Hypothesis 2:* Psychological capital is positively related to knowledge collecting (a) and knowledge donating (b), and the influence of psychological capital on knowledge donating is stronger than that on knowledge collecting.

### Knowledge Sharing and IWB

Making full use of internal existing knowledge is an effective way to create new knowledge. However, the internal knowledge of the organization is always distributed in different departments and individuals. Only through the sharing and exchange of different departments and employees can these scattered knowledge flow fully within the organization, so as to promote the integration of different knowledge (Nerkar and Roberts, 2004) and then create new knowledge. Knowledge sharing can promote the accumulation and flow of knowledge, which is essential for the translation of individual knowledge to organizational knowledge (Hooff and Ridder, 2004) and the generation of new knowledge. Therefore, it is positively related to employees’ creative behavior, innovation capability, and innovation performance (Kim and Lee, 2013; Akhavan and Hosseini, 2016; Pian et al., 2019; Clercq and Pereira, 2020; Ganguly et al., 2020), which is also an important factor affecting employees’ IWB (Akram et al., 2020; Asurakkody and Kim, 2020; Yasir et al., 2021). As mentioned above, knowledge sharing consists of two central processes: knowledge collecting and donating (Hooff and Ridder, 2004). They are also closely related to employee innovation. Due to different research premises and research objects, in the existing research, scholars hold different views on the role of knowledge collecting and knowledge donating as mediating mechanisms on employee innovation (Kim and Lee, 2013; Kmieciak, 2020). Because of the transformative and complex nature of innovation, it is usually difficult for individuals to possess all of the knowledge required to solve problems. Knowledge collecting can help to improve the breadth and depth of individual knowledge bases, which is conducive to the expansion and update of individual knowledge (Akhavan and Hosseini, 2016; Chatterjee et al., 2020), and this will create favorable conditions for individual innovation. Knowledge donating is a process of sharing one’s intellectual capital with others (Hooff and Weenen, 2004), in which individuals pay more than they gain. Therefore, it is difficult to directly support individual innovation and may even expose individuals to the risk of losing their unique advantages. Hence, our third hypothesis is as follows:

*Hypothesis 3:* Knowledge collecting (a) is positively related to IWB, while knowledge donating (b) is negatively related to IWB.

### The Mediating Roles of Knowledge Collecting and Donating

Employees’ psychological capital needs to be turned into action which is beneficial to improve innovation to have better result. The positive mental state of employees with high psychological capital represents a stable resource. They are more likely to maintain an optimistic, confident, and hopeful state, do not easily give up in the face of difficulties and setbacks, and can quickly recover from failure and find solutions to problems. These traits will effectively eliminate the negative feelings that employees may experience regarding knowledge sharing (Philipp et al., 2019; Lines et al., 2020), maintain, and enhance their willingness to collect and donate knowledge, and ultimately play an indirect and significant role in promoting IWB. However, due to different connotations and properties, knowledge collecting and knowledge donating may have correlation and different mediating effects, which is rarely concerned by research at present.

SET will provide support for understanding the relationship between knowledge collecting and knowledge donating and their specific mediating roles. SET holds that the process of social communication can be said to be an exchange process. Those who can provide us with the most benefits are the people who are most attractive to us, and in order to get benefits, we also have to pay benefits. The essence of knowledge sharing is the social exchange of knowledge, which is a two-way process. A harmonious and effective knowledge sharing system necessitates the coexistence of knowledge collecting and donating (Zhou et al., 2013). The question we strive to answer is, in the continuous social exchange, are there any differences and correlations between the influences of knowledge collecting and donating on IWB?

Based on the reciprocity norm of SET (Gouldner, 1960), during knowledge sharing, when other organization members benefit from knowledge donating by employees, these members will also be more willing to donate their own knowledge. Therefore, in the social exchange process of knowledge sharing, the knowledge donating of employees will help to improve the willingness of other organization members to participate in knowledge sharing and be conducive to the creation of a good organizational communication atmosphere. A constructive communication atmosphere is a core requisite for successful knowledge sharing (Moffett et al., 2003; Hooff and Ridder, 2004). In addition, knowledge donating facilitates positive interactions between individuals and organizations, which promote the formation of good interpersonal relationships and interaction environments and create favorable conditions for individual knowledge collecting, that is, individuals who often participate in knowledge donating are more likely to obtain knowledge donated by other organization members. Wang and Luo (2011) concluded that knowledge donating can help knowledge donors gain respect and a good reputation and that these intangible rewards are drivers of knowledge sharing. While donating knowledge, knowledge donors will also receive the reception of either knowledge feedback or similar responses and rewards in the future (Zhu et al., 2019), making knowledge donors become knowledge collectors. Therefore, knowledge donating may indirectly affect IWB by promoting knowledge collecting. Hence, we put forth the following hypothesis:

*Hypothesis 4a:* Knowledge donating is positively related to knowledge collecting, and they play a chain mediating role in the relationship between psychological capital and IWB.

Similarly, based on the reciprocity norm of SET, when employees perceive the existence of social exchange with their organization, they will strengthen the social emotional bond with the organization, which is specifically reflected in the improvement of emotional commitment, task performance, and OCB (Song et al., 2009). Therefore, when employees acquire knowledge from other organization members and improve their own knowledge reserves, they will also believe that they have the obligation to repay others with similar behaviors, thereby strengthening knowledge donating behaviors. We therefore propose another hypothesis:

*Hypothesis 4b:* Knowledge collecting is positively related to knowledge donating, and they play a chain mediating role in the relationship between psychological capital and IWB.

We proposed that knowledge collecting and knowledge donating are closely related to psychological capital and IWB. Knowledge collecting and knowledge donating together constitute knowledge sharing. As the two central processes of knowledge sharing, in addition to the chain mediating effect of their interactions, they may also play a parallel mediating role. Therefore, We further propose the following hypothesis:

*Hypothesis 4c:* Knowledge collecting and knowledge donating play a parallel mediating role in the relationship between psychological capital and IWB.

### The Moderating Role of Age

The relationship of psychological capital and knowledge collecting/knowledge donating is different between individuals. The strength of the relationship between them may be affected by certain factors. Age is generally regarded as an important factor affecting employees’ psychological state and workplace behavior (Jirsaraie et al., 2019; Anser et al., 2020; Sammarra et al., 2021), and studies have proved the moderating effect of age in the relationship between individual psychology factors and behavior (Meyers et al., 2020; Okumah et al., 2020). We included age as a boundary condition in the study to explore the difference of the impact intensity of psychological capital on knowledge collecting and knowledge donating among employees of different ages.

Employees’ values, work motivation, and personality will change with age (Labouvie-Vief et al., 2000). Employees of different ages will have different reactions in the workplace due to changes in values and motivation (Kanfer and Ackerman, 2004). Individual personality characteristics encompass maturity and cumulative continuity principles, that is, with age, individual personality characteristics tend to stabilize (Jenny et al., 2019; Mann et al., 2019). Younger employees will respond more positively to any inducement conducive to their career aspirations and knowledge acquisition, while older employees prefer stability and obedience (Wu et al., 2018; Scheibe, 2021).

For older employees, on the one hand, after a long-term accumulation, they have completed the initial accumulation of intellectual capital and self-realization, so the motivation and behavior pattern of knowledge collecting will be more stable. On the other hand, they usually pay more attention to maintaining existing interests (Thomas and Anderson, 1998; Tordera et al., 2020), prefer to adopt moderate or conservative strategies for action, and believe that obeying the organization’s arrangement is more important than actively striving for it (Labouvie-Vief et al., 2000; Wu et al., 2018), which makes the knowledge donating of older employees need more external stimulation rather than psychological influence. In contrast, younger employees, who are in the early stages of career growth, usually pay more attention to the attainment of goals or optimal performance (Freund, 2006), believe that they can create better development opportunities by acquiring, practicing, and improving skills and resources related to their goals, driving by an urgent need to use their own initiative to achieve success (Ebner et al., 2006). These characteristics are easier to increase the urgency of younger employees’ knowledge sharing demands, urge them to pay more attention to and measure the collecting and donating of knowledge, and strengthen the impact of their psychological state on behavior.

Therefore, we speculate that age may be an important moderating factor. The knowledge collecting/donating behavior of older employees are less affected by psychological capital, whereas those of younger employees are more affected by psychological capital:

*Hypothesis 5:* Age moderates the relationship between psychological capital and knowledge collecting (a) /knowledge donating (b), that is, the higher the age, the weaker the correlation between psychological capital and knowledge collecting (a) /knowledge donating (b), and vice versa.

## Materials and Methods

### Participants and Procedures

In the formal survey, the respondents were regular employees from Chinese cities (Shanghai, Beijing, Guangzhou, Wuhan, etc.) engaged in information technology (IT), testing and certification, research and development (R&D), management, and other sectors. Data were collected through QQ, WeChat, e-mails, on-site questionnaires, and other media. Two scales were collected for each respondent: the survey on psychological capital, knowledge sharing, and demographic information, which was completed by the employees, and the employee IWB evaluation scale, which was completed by their leaders. Independent coding was adopted to ensure matching between employees and leaders. In this survey, 420 questionnaires were distributed and 377 were collected (response rate: 89.76%), of which 345 were deemed valid (effective response rate: 82.14%).

Of the 345 valid respondents, 64.6% were male and 35.4% were female, 62.3% were married and 37.7% were unmarried. The ages of participants were divided into different age groups as follows: under age 25 (44.3%), 26–30years old (27.5%), 31–40years old (12.2%), and 41years old and above (15.9%). Moreover, 6.9% of participants were educated to senior middle school and below, 21.2% to junior college level, 55.7% to bachelor degree level, and 16.2% to master degree level and above. With respect to job tenure, 23.2% of participants had worked for their organization less than 1year, 37.4% had between 1 and 5years of job tenure, 14.2% had between 5 and 10years, and 25.2% had more than 10years.

### Measures

Employees responded to questions regarding psychology capital, knowledge sharing, and IWB on a 5-point scale, from 1 (completely disagree) to 5 (completely agree). Given that our samples were Chinese, the double-blind back-translation procedure (Schaffer and Riordan, 2003) was utilized to translate all items into Chinese. To facilitate understanding, each item was translated by professional translators.

#### Psychological Capital

The psychological capital scale compiled by Luthans et al. (2003) consists of four dimensions: self-efficacy, hope, optimism, and resilience, with 24 items in total. Some representative items are “I believe that I have the ability to analyze and solve problems” and “I am energetic enough to achieve my work goals” (*α*=0.962).

#### Knowledge Collecting and Knowledge Donating

The 10-item scale developed by Hooff and Weenen (2004) was employed to assess the two central processes of knowledge sharing. Knowledge collecting includes 4 items. The representative item is “Colleagues within my department tell me what they know when I ask them about it” (*α*=0.914); Knowledge donating includes 6 items. The representative item is “When I have learned something new, I tell my colleagues in my department about it” (*α*=0.910).

#### Innovative Work Behavior

A 9-item scale to measure IWB was adopted from the work of Janssen (2000). This scale has three dimensions: the generation, promotion, and realization of innovation ideas. Some representative items are “I can often create new ideas for difficult issues” and “I always search out new working methods, techniques, or instruments” (*α*=0.955).

We also designated gender (1=*male*, 0=*female*), marital status, education level, and number of working years as control variables.

### Data Analysis

SPSS v26.0 and AMOS v21.0 software was used for the internal consistency, factor analysis, common method variance test, descriptive statistics, and correlations among the variables. In addition, for hypothesis 1–4, we used the SPSS macro PROCESS to test. Taking psychological capital as the antecedent variable, knowledge collecting, and knowledge donating as the mediating variable and IWB as the outcome variable, we conducted Bootstrap sampling after adding the control variable and repeatedly sampled 5,000 samples to calculate the 95% confidence interval (CI). We then observed whether the CI of each path contains zero to evaluate whether the mediating effect is significant. Similarly, the PROCESS macro was also used to test the moderating effect of age. The subjects were divided into high/low age groups, and a simple slope plot was used to estimate the impact of psychological capital on knowledge collecting/knowledge donating in high/low age groups to test hypothesis H5.

## Results

### Factor Analysis

For the pilot survey, the questionnaire was revised for one round and was found to have good content validity. Therefore, the next step was to conduct the Kaiser–Meyer–Olkin (KMO) test and Bartlett test of sphericity on the sample data to test the structural validity. The KMO values of the psychological capital (0.963), knowledge collecting (0.742), knowledge donating (0.841), and IWB (0.950) scales were either greater than or close to 0.9, and the values of *p* Bartlett test were lower than the significance level of 0.001, indicating that the three variables were suitable for factor analysis. In addition, the standard factor loading of each variable was between 0.612 and 0.910. The average variance extraction of each variable was >0.5, indicating that the variables in this study had good convergence validity and that the research model fit well.

### Common Method Variance Test

According to the research of Podsakoff et al. (2003), we used the unmeasurable potential method factor effect control to test the common method variance.

The results showed that the goodness-of-fit index of the model before adding method factor is: *χ*^2^/df=3.844, *p*<0.001; CFI=0.837; TLI=0.826; RMSEA=0.091; SRMR=0.0586. Although the fitting effect of the data is not ideal, the CFA test results showed that the model that allowed the various items to load onto their respective factors produced a better model fit than any of the models in which the scales were combined. Therefore, the four-factor model produced the best model fit for our data. After adding the method factor (*χ*^2^/df=3.091, *p*<0.001; CFI =0.888; TLI=0.872; RMSEA=0.078; SRMR=0.0477), according to the measurement standards proposed by Wen et al. (2018), the fit of the model has not been greatly improved (ΔCFI =0.051, ΔTLI=0.046; ΔRMSEA=0.013; ΔSRMR=0.0109). Therefore, it can be judged that the common method variance is not serious in this study.

### Descriptive Statistics

Table 1 presents means, standard deviations, and bivariate correlations for all study variables, which provide preliminary support for subsequent hypothesis testing.

**Table 1:** Descriptive statistics and correlations among all variables.

Variables	*M*	*SD*	1	2	3	4	5	6	7	8	9
1. Gender	–	–	1								
2. Marital status	–	–	0.062	1							
3. Education	3.81	0.859	0.030	−0.221^***^	1						
4. Job tenure	2.72	1.516	0.134^*^	0.809^***^	−0.269^***^	1					
5. Age	3.06	1.238	0.152^**^	0.757^***^	−0.132^*^	0.842^**^	1				
6. Psychological capital	3.80	0.712	0.115^*^	0.114^*^	−0.060	0.152^**^	0.138^*^	1			
7. Knowledge collecting	3.68	0.884	−0.026	0.041	−0.087	0.025	−0.010	0.643^***^	1		
8. Knowledge donating	3.67	0.861	0.009	0.093	−0.071	0.091	0.058	0.688^***^	0.732^***^	1	
9. IWB	3.74	0.816	0.169^**^	0.033	0.005	0.064	0.055	0.831^***^	0.661^***^	0.648^***^	1

*N*=345. Gender: 1=male, 0=female; Marital status: 1=married, 2=unmarried; Education: 1=senior middle school and below, 2=junior college, 3=bachelor degree, 4=master degree, 5=doctoral degree and above; Job tenure: 1=under 1year, 2=1–5years, 3=5–10years, 4=above 10years; Age: 1=under age 25, 2=26–30-year-old, 3=31–40-year-old, 4=41–50-year-old, 5=51–55-year-old, 6=56-year-old and above.

* *p* <0.05;

** *p* <0.01;

*** *p* <0.001.

### Mediating Effect of Knowledge Collecting and Knowledge Donating

The multicollinearity test showed that the tolerance of all of the predictive variables was between 0.325 and 0.971 (> 0.10), the variance expansion factor was between 1.03 and 3.08 (< 10), so the model did not have a multicollinearity problem. To determine the specific mechanism by which knowledge collecting and donating mediate the relationship between psychological capital and IWB, we used the PROCESS macro after adding control variables.

**Figure 1:**
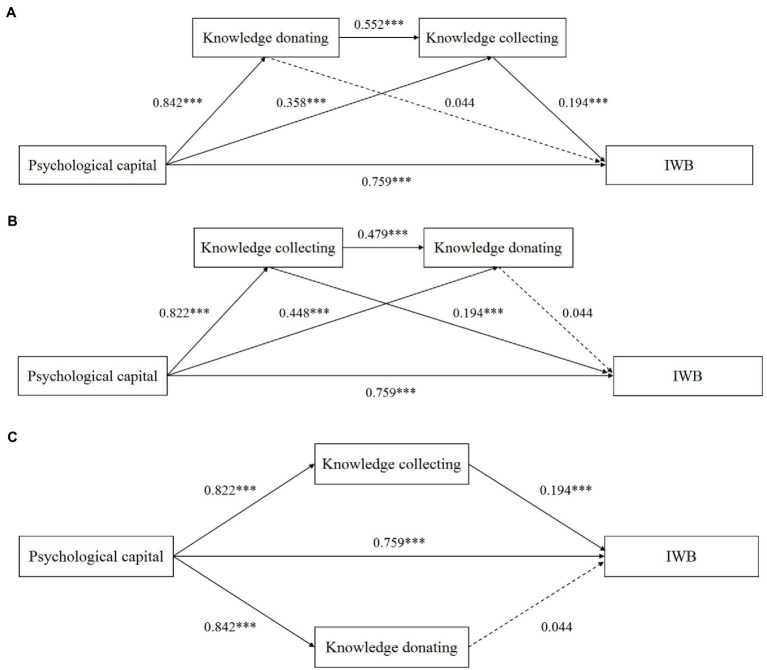
Competitive models of the mediating effects of knowledge collecting and knowledge donating. **(A)** Knowledge donating and knowledge collecting play a chain mediating role in the relationship between psychological capital and IWB. **(B)** Knowledge collecting and knowledge donating play a chain mediating role in the relationship between psychological capital and IWB. **(C)** Knowledge collecting and knowledge donating play a parallel mediating role in the relationship between psychological capital and IWB. Competitive models of the mediating effects of knowledge collecting and knowledge donating. Dashed lines indicate that the path is not significant. ^***^p < 0.001.

Figure 1 showed the path coefficients of the three competitive models. In Figure 1A which the chain mediating path was verified, the direct impact of psychological capital on IWB was significant (*B*=0.759, *p*<0.001), which supported H1. Psychological capital was positively related to knowledge collecting (*B*=0.358, *p*<0.001) and donating (*B*=0.842, *p*<0.001), supporting H2a and H2b. In addition, according to the impact of psychological capital on knowledge collecting and knowledge donating when they play a parallel mediating role in Figure 1C, it can be found that the correlation between psychological capital and knowledge donating (*B*=0.842, *p*<0.001) is stronger than that with knowledge collecting (*B*=0.822, *p*<0.001). There was a weak gap between them, which further supported H2, that is, the influence of psychological capital on knowledge donating is stronger than that on knowledge collecting. Finally, knowledge collecting was positively related to IWB (*B*=0.194, *p*<0.001), supporting H3a. The relationship between knowledge donating and IWB was not significant (*B*=0.044, *n.s*.), implying that H3b was not supported.

According to the mediating effect test results: Table 2A showed that psychological capital has a significant indirect impact on IWB through knowledge donating and knowledge collecting (effect=0.090, 95% CI: [0.043, 0.140]), supporting H4a; Table 2B showed that the indirect impact of psychological capital on IWB through knowledge collecting and knowledge donating is not significant (effect=0.017, 95% CI: [−0.021, 0.064]), which does not supporting H4b; Table 2C showed that the indirect impact of psychological capital on IWB through knowledge collecting is significant (effect=0.160, 95% CI: [0.078, 0.240]), while the indirect impact of psychological capital on IWB through knowledge donating is not significant (effect =0.037, 95% CI: [−0.048, 0.131]), which does not support H4c. Therefore, we believe that knowledge donating is positively related to knowledge collecting, and they play a chain mediating role between psychological capital and IWB.

**Table 2:** Chain mediating effect analysis.

Model pathways	Estimated effect	*SE*	Bootstrap 95% Confidence Interval	
			Lower bounds	Upper bounds
**(A)**				
Psychological capital→Knowledge collecting→IWB	0.070	0.022	0.030	0.117
Psychological capital→Knowledge donating→IWB	0.037	0.047	−0.047	0.131
Psychological capital→Knowledge donating→Knowledge collecting→IWB	0.090	0.024	0.043	0.140
Direct effects: Psychological capital→IWB	0.759	0.047	0.665	0.852
**(B)**				
Psychological capital→Knowledge collecting→IWB	0.160	0.041	0.076	0.236
Psychological capital→Knowledge donating→IWB	0.020	0.025	−0.026	0.073
Psychological capital→Knowledge collecting→Knowledge donating→IWB	0.017	0.022	−0.021	0.064
Direct effects: Psychological capital→IWB	0.759	0.047	0.665	0.852
**(C)**				
Psychological capital→Knowledge collecting→IWB	0.160	0.041	0.078	0.240
Psychological capital→Knowledge donating→IWB	0.037	0.046	−0.048	0.131
Direct effects: Psychological capital→IWB	0.759	0.047	0.665	0.852

### Moderating Effect of Age

**Figure 2:**
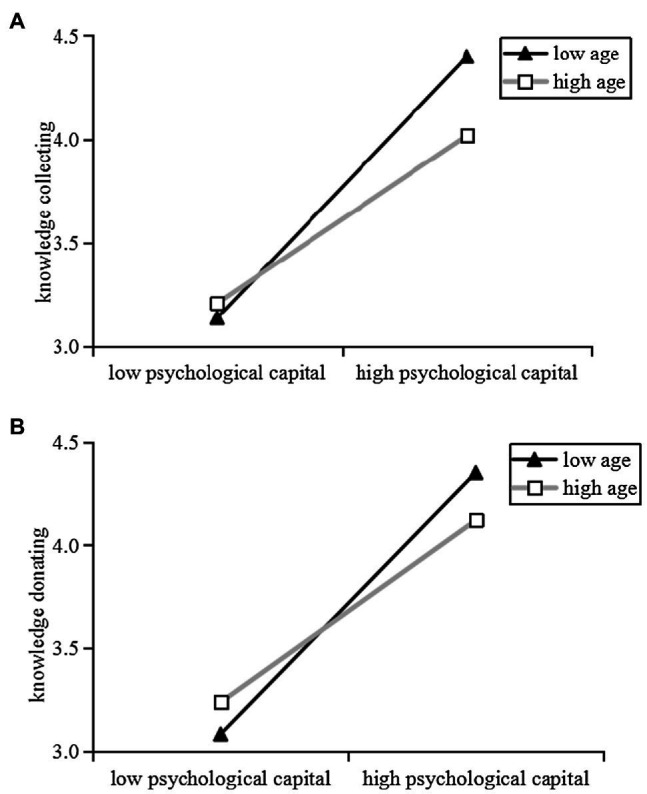
Moderating effect of age. **(A)** Moderating effect of age on the relationship between psychological capital and knowledge collecting. **(B)** Moderating effect of age on the relationship between psychological capital and knowledge donating.

Finally, the moderating effect of age was tested. The results in Table 3 indicated that the psychological capital × age interaction term was significantly related to knowledge collecting (*B*=−0.126, *p*<0.05) and knowledge donating (*B*=−0.106, *p*<0.05). To more directly assess the moderating effect of age, a simple slope plot was used (see Figure 2). The simple slope analyses showed that the effects of psychological capital on knowledge collecting and knowledge donating were stronger for lower age than for higher age (knowledge collecting – lower age: *B*=0.933, *p*<0.001, higher age: *B*=0.650, *p*<0.001; knowledge donating – lower age: *B*=0.927, *p*<0.001, higher age: *B*=0.595, *p*<0.001). There was a significant difference in the correlation between psychological capital and knowledge collecting/knowledge donating between high age group and low age group, which supported H5a and H5b.

**Table 3:** Moderating effect analysis.

Dependent variable	Knowledge collecting	Knowledge donating	Model 1	Model 2
Constant	0.425	0.142
**Control Variable**
Gender	−0.148^*^	−0.109
Marital status	0.158	0.144
Education level	−0.062	−0.026
Working years	−0.030	−0.002
**Independent Variable**
Psychological capital	1.187^***^	1.149^***^
Age	−0.069	−0.050
Psychological capital ^*^ Age	−0.126^*^	−0.106^*^
*R* ^2^	0.451	0.493
*F*	39.528	46.851

* *p* <0.05;

*** *p* <0.001.

**Figure 3:**
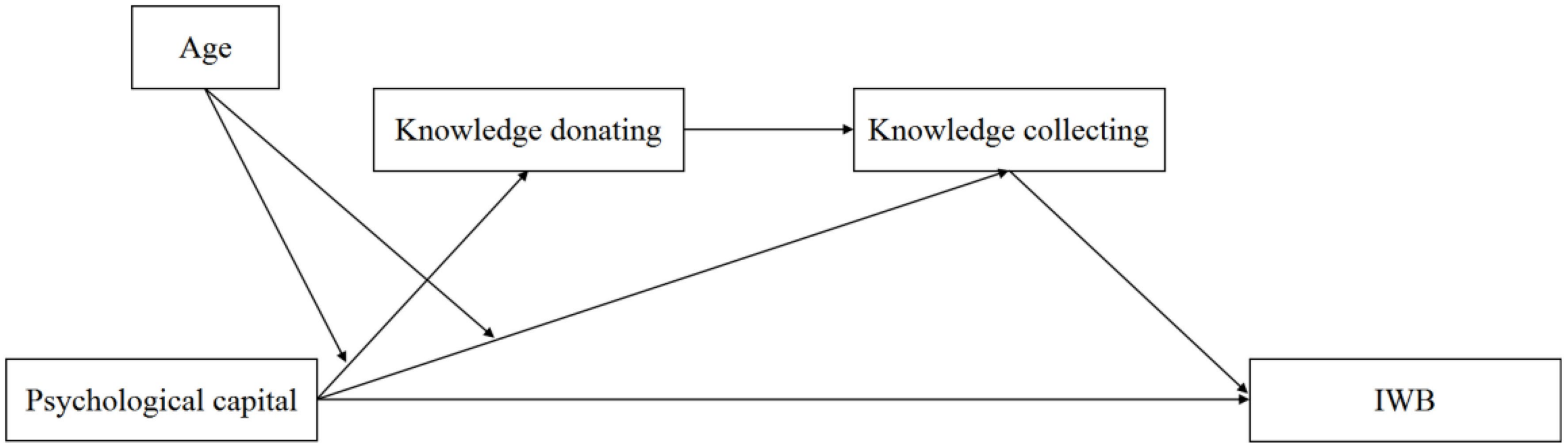
The final model.

The final model obtained is shown in Figure 3.

## Discussion

The impact of employees’ psychological capital on IWB has attracted the attention of many scholars (Guo et al., 2019; Tang et al., 2019; Gao et al., 2020; Yan et al., 2020), however, the research of their relationship from the perspective of the two central processes of knowledge sharing (knowledge collecting and donating) has not been discussed. Through the analysis of three competitive models, we found that knowledge donating and knowledge collecting play a chain mediating role in this process.

The present study confirmed that employees’ psychological capital is positively related to their IWB and knowledge sharing (knowledge collecting and donating), which is consistent with previous study results (Qiu et al., 2015; Wu and Lee, 2017; Zhang et al., 2017; Guo et al., 2019; Tang et al., 2019; Gao et al., 2020; Yan et al., 2020). Psychological capital that help to maintain one’s open and stable mentalities is important for driving employees to participate in knowledge sharing. It can also help individuals overcome the possible negative effects of knowledge donating, make them recover from setbacks faster, give employees positive psychological strength to resist negative effects and promote knowledge donating. Furthermore, we also found that psychological capital has a stronger impact on knowledge donating. Thus, we believe that although psychological capital helps employees actively participate in communication and better integrate into the organization, due to the potential risk of losing the advantages in the process of knowledge donating, knowledge donating may need the support of psychological capital more.

Moreover, we found the mediating roles of knowledge collecting and knowledge donating between employees’ psychological capital and IWB includes two paths: the single mediating role of knowledge collecting and the chain mediating role of both knowledge donating and collecting; that is, knowledge collecting has a significant direct impact on IWB, whereas knowledge donating does not directly affect IWB, but indirectly influences it by promoting knowledge collecting. Knowledge collecting can enable employees to expand their personal knowledge reserve, enrich the knowledge required for innovation and enhance their own ability, which has an obvious gain effect on IWB (Smith, 2006). Although from the perspective of employees, knowledge donating may not affect or even be disadvantageous to IWB, our empirical analysis provides different results. In fact, knowledge donating has an indirect positive impact on IWB. According to the SET, knowledge donating helps individuals cultivate positive interpersonal interaction, form good reputation image, and better integrate into organizational knowledge sharing activities, so as to provide channels and opportunities for individual knowledge collecting and then promote IWB.

Another finding is the moderating effect of age on the relationship between psychological capital and knowledge collecting/knowledge donating. With the increase of age, individual personality characteristics tend to be stable (Jenny et al., 2019; Mann et al., 2019), which will form a relatively stable behavior pattern and social style. Therefore, the correlation between psychological capital and knowledge collecting/knowledge donating is weakened by the age.

### Theoretical Implications

Our research makes several contributions to theory. First, we expand the body of traditional knowledge sharing research and elucidate the modes of influence of the two central processes of knowledge sharing. Although the existing studies have distinguished knowledge collecting and knowledge donating, they have seldom observed or analyzed the correlation between them and their respective influence mechanisms, especially the specific impact of knowledge donating on individuals. We have made a more in-depth analysis on this. More specifically, our results have attracted attention to the potential “bright” side of knowledge donating, promoting a more balanced theoretical perspective of knowledge sharing by highlighting the two central processes that previous research has ignored.

The present investigation expands our knowledge on the impact mechanism associated with IWB. Our results reaffirm the importance of integrating psychological capital and knowledge sharing into the study of IWB and creatively explore the relationship between psychological capital and IWB from the perspective of knowledge aggregation and flow, which is a supplement and expansion of the existing theoretical framework. Innovation is the process of realizing the value addition of knowledge in the flow and concentration of knowledge. Paying attention to the flow state of knowledge is a more in-depth and closer to the essence of IWB.

Furthermore, our results highlight a key boundary condition for the role of psychology capital. Previous studies confirmed the influence of psychological capital on knowledge sharing (Qiu et al., 2015; Wu and Lee, 2017; Zhang et al., 2017), but the boundary conditions of this relationship were ill-defined. We found that the correlation between psychological capital and knowledge collecting/knowledge donating differs with age. As such, to fully understand the consequences of psychological capital and knowledge sharing, it appears important to consider his or her age as a key contingency factor. These findings further expand our understanding of psychological capital and knowledge sharing, provide novel insights into a variety of employee attitudes and behaviors, and add value to our understanding of employee and organizational phenomena.

### Practical Implications

Our study provides several practical implications for organizations. First, our research verifies that psychological capital can trigger a series of positive behaviors, having positive impact on knowledge sharing and IWB. Therefore, we recommend that organizations should pay attention to the psychological capital and mental health of employees.

Secondly, knowledge sharing is considered one of the most important aspects of knowledge management (Zhang and Jiang, 2015; Obrenovic et al., 2020), and knowledge donating is the one of the most difficult aspects of knowledge sharing. We address a question posed by many knowledge workers on the significance of knowledge donating on individual innovation and confirm the positive impact of knowledge donating at the individual level. In reality, organizations also need to face this question and carry out effective training for employees so that they understand that knowledge donating can also indirectly bring feedback to their growth and development. Having invariable knowledge is not the core competitiveness of individuals, constantly creating and using knowledge is the foundation of personal development. At the same time, the organization should establish an open and mutual cultural atmosphere to reduce the sense of threat brought by knowledge donating, make employees feel that sharing knowledge is safe and successful, and stimulate employees’ willingness to participate in knowledge donating.

As knowledge collecting directly affects IWB, organizations also need to create favorable conditions for knowledge collecting by employees. Organizations should establish a knowledge sharing system and all-round incentive system both online and offline to allow employees to easily access help and solutions, understand job-related knowledge, and realize self-display and self-growth.

A final implication for organizations concerns the role that age plays in this process. Organizations should provide targeted incentives to employees of different ages. Research shows that knowledge sharing in younger employees is more easily affected by psychological capital. Organizations should pay more attention to the psychological capital of younger employees, conduct timely guidance, and offer adequate incentives. For older employees, organizations should adapt and try a variety of incentive policies to find the focus to stimulate their willingness to participate in knowledge sharing.

### Limitations and Future Research

This study inevitably had some limitations. First, all of our samples were collected from China; the universality of the relationship among psychological capital, knowledge sharing, and IWB needs to be verified in different regional cultural backgrounds. In accordance with the recommendation of Marôco (2010), researchers on future should test our model with distinct samples and in more diverse industries to allow greater generalization.

Second, the nature of our study design was cross-sectional. We collected knowledge collecting and knowledge donating data at a single time point according to the initial design of the knowledge sharing scale. We suppose that knowledge collecting and donating as individual behavior habits do not change significantly over short periods of time, but our claim of causality is not convincing enough. In the future, it is necessary to study the dynamics of variable interactions and adopt longitudinal research methodologies for long-term follow-up surveys.

Third, the measurement of psychological capital, knowledge collecting, and knowledge donating relied on employees’ self-report, which may lead to common method variance. We believe that because these variables reflect employees’ psychological feelings and attitudes, employees may be more accurate and reliable informants. However, if data can be collected from multiple sources and be controlled or compared during analysis in the future, more reasonable and effective results may be obtained.

Fourth, the factors that affect IWB are complex and diverse. For instance, we did not consider the influence of the organizational level on IWB. Future research should either introduce organizational factors into the model or expand the model to the organizational level to explore other mechanisms by which IWB is affected.

Fifth, our research takes employees engaged in professional and technical work as the sample, so the results may be more applicable in knowledge-based and technological enterprises with knowledge accumulation, while the adaptability to production-oriented, trade-oriented or service-oriented enterprises remains to be verified.

Finally, the influence of knowledge donating on IWB may also be affected by other factors, such as the organization type and position type of employees. The status of donors, such as ordinary employees, core members, and experts with authority and leaders, may also influence the effects of knowledge donating on their own innovation abilities. In addition, the content and types of knowledge are complex and diverse, such as empirical knowledge, common sense knowledge, technical knowledge, monopoly knowledge. Various types of knowledge involved in knowledge donating may have different effects on employees’ willingness to share knowledge and personal innovation. In the future, we can consider the influence of these factors and conduct more detailed and comprehensive studies on the relationship between knowledge donating and IWB.

## Conclusion

From the perspective of knowledge sharing, the present study deeply analyzes the psychological processing mechanism and boundary conditions of psychological capital on IWB and confirms the positive significance of knowledge donating at the individual level. On the basis of previous studies, we conducted an in-depth study of the two central processes of knowledge sharing, knowledge collecting, and donating and explored their mutual relationship and the mechanisms by which they influence IWB. According to the empirical analysis, we found that psychological capital can affect IWB through knowledge collecting and can also indirectly affect IWB through the chain mediating role of knowledge donating and collecting. In addition, age was found to play a moderating role in the relationship between psychological capital and knowledge sharing, and the relationship between them decreased as age increased. Our findings verify the differences and correlations between knowledge collecting and donating as the central processes of knowledge sharing, directly face the grievances of most employees who are unwilling to participate in knowledge donating, and prove the positive significance of knowledge donating to the donors themselves. Knowledge donating does not have a direct impact on the IWB of employees, but can promote IWB by promoting knowledge collecting. Thus, our study provides a new perspective for organizations to effectively promote knowledge sharing and stimulate IWB in the future.

## Data Availability Statement

The raw data supporting the conclusions of this article will be made available by the authors, without undue reservation.

## Author Contributions

WC designed and drafted this work. XZ participated in the paper writing and analyzed data. SS, SL, and ZG have made a substantial, direct, and intellectual contribution to the work. All authors contributed to the article and approved the submitted version.

## Conflict of Interest

The authors declare that the research was conducted in the absence of any commercial or financial relationships that could be construed as a potential conflict of interest.

## Publisher’s Note

All claims expressed in this article are solely those of the authors and do not necessarily represent those of their affiliated organizations, or those of the publisher, the editors and the reviewers. Any product that may be evaluated in this article, or claim that may be made by its manufacturer, is not guaranteed or endorsed by the publisher.
